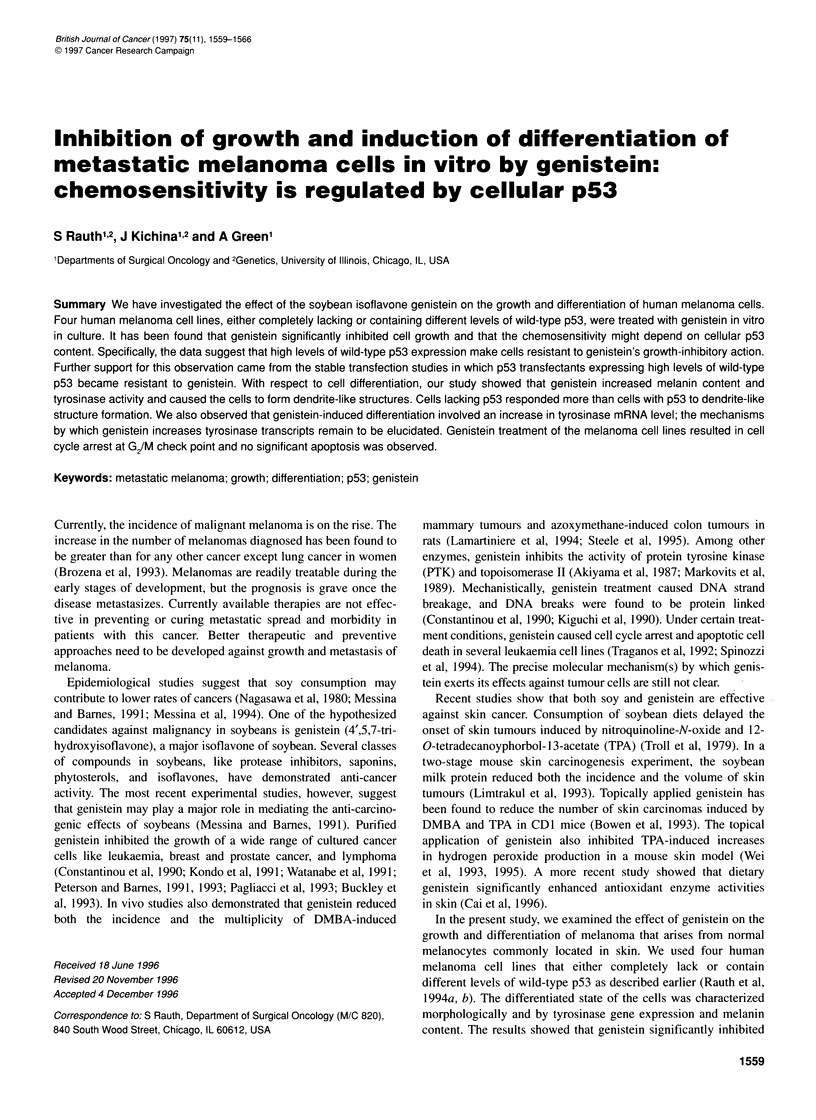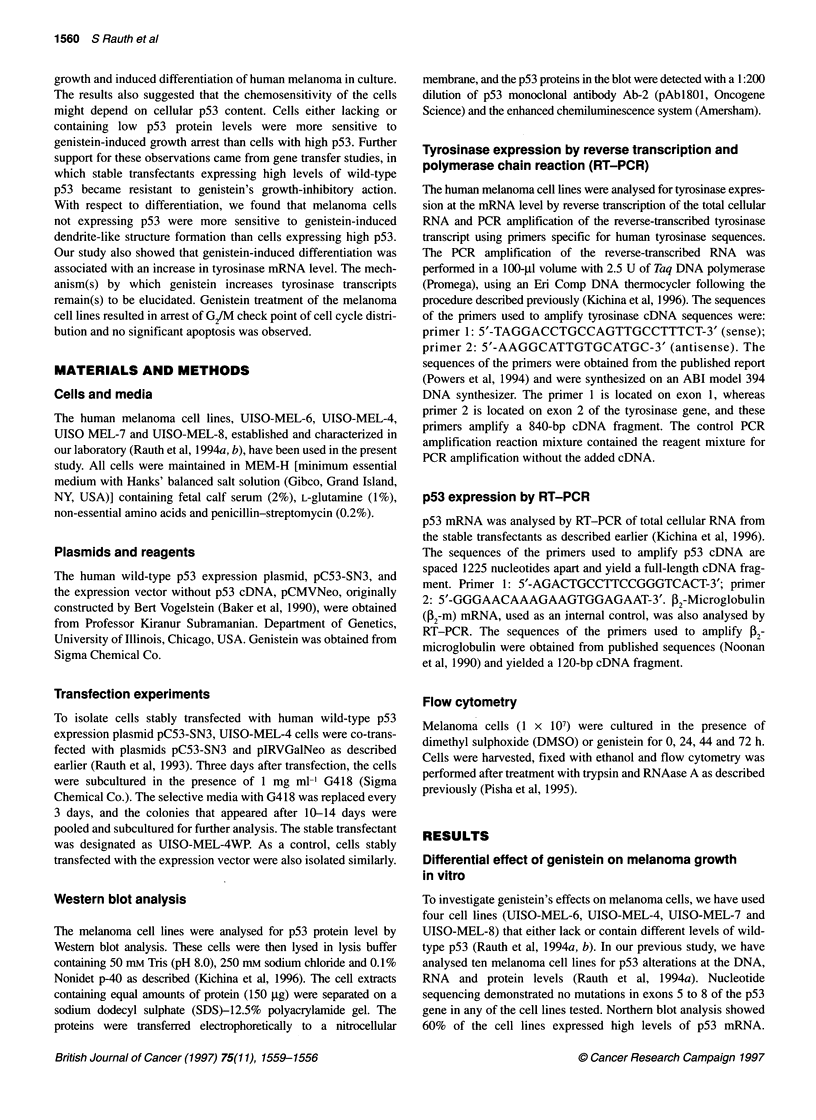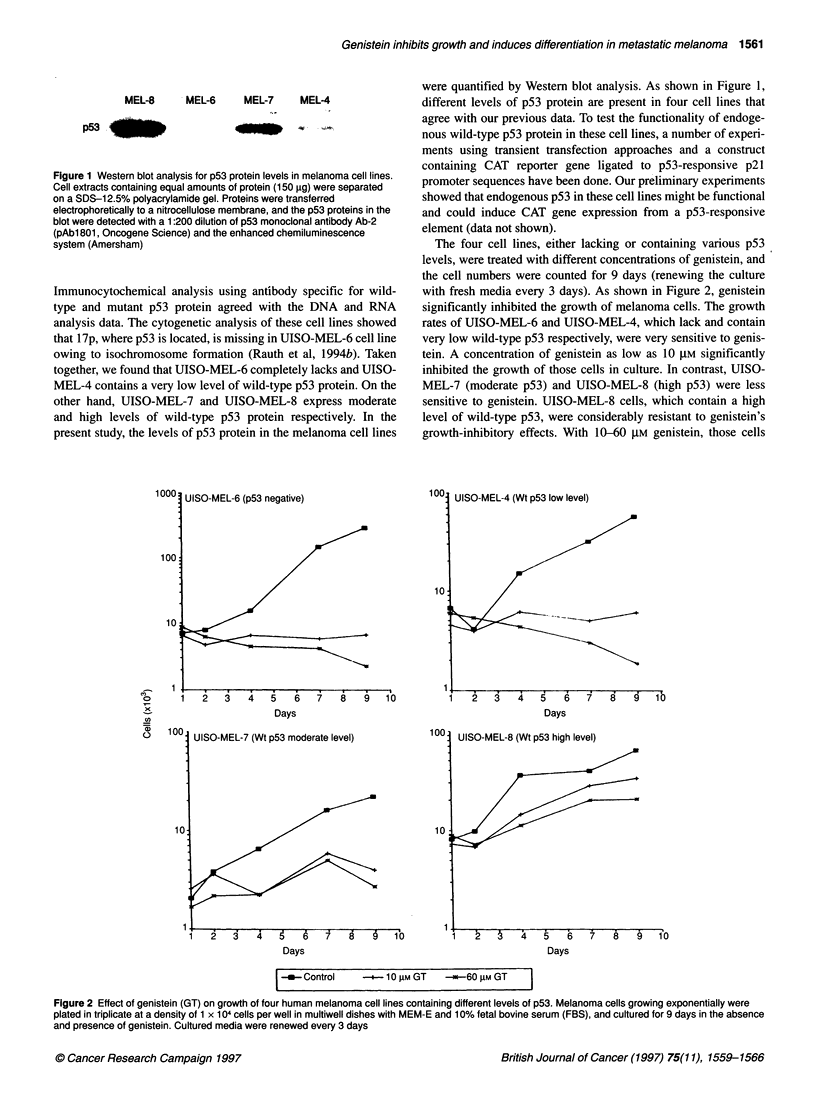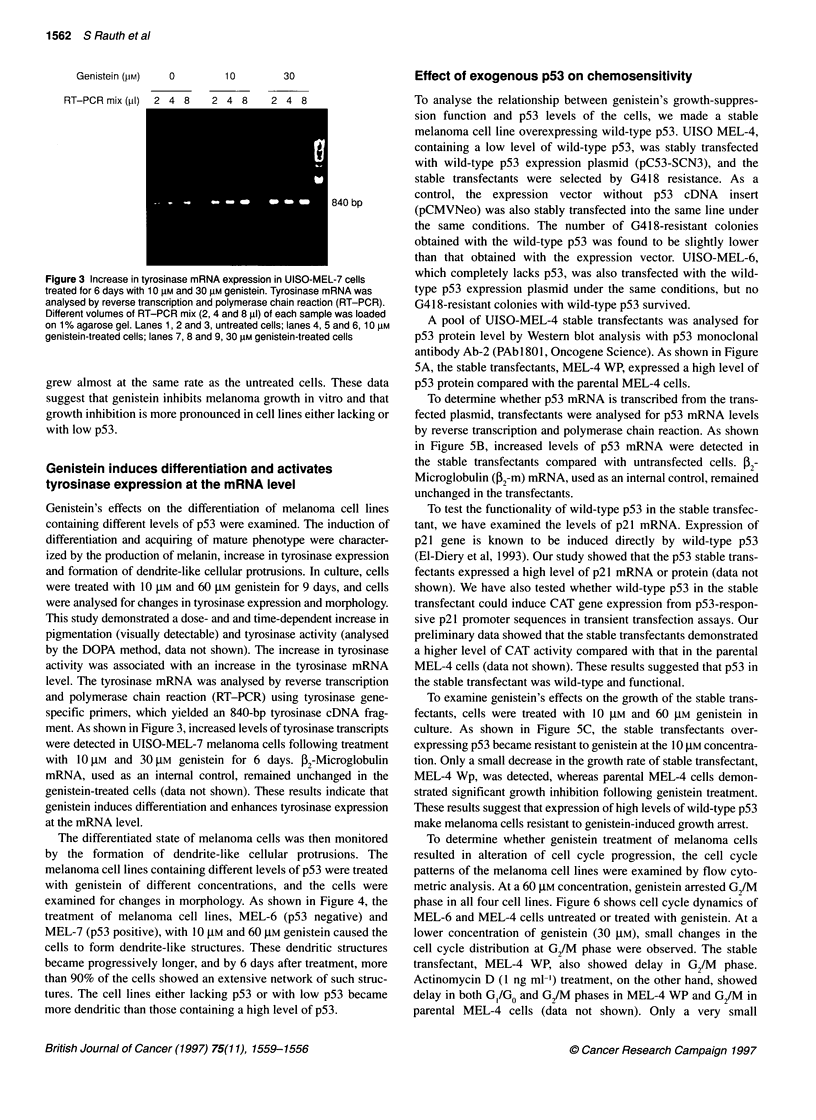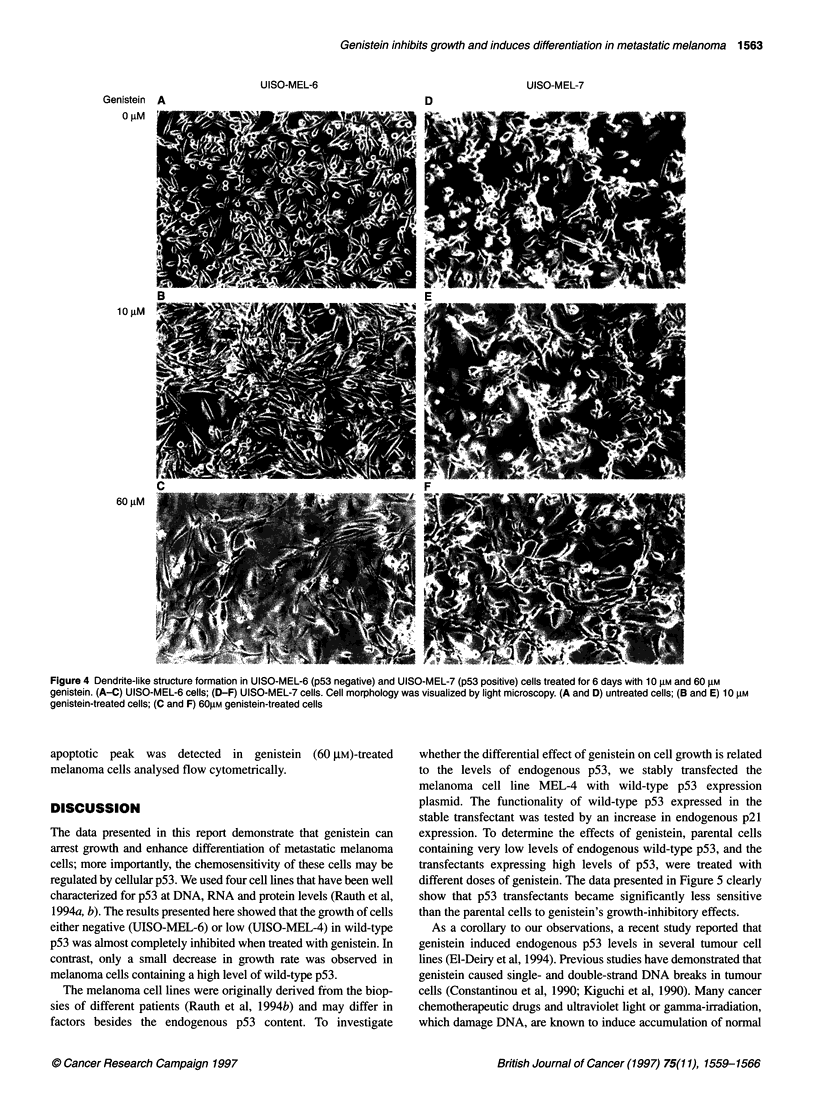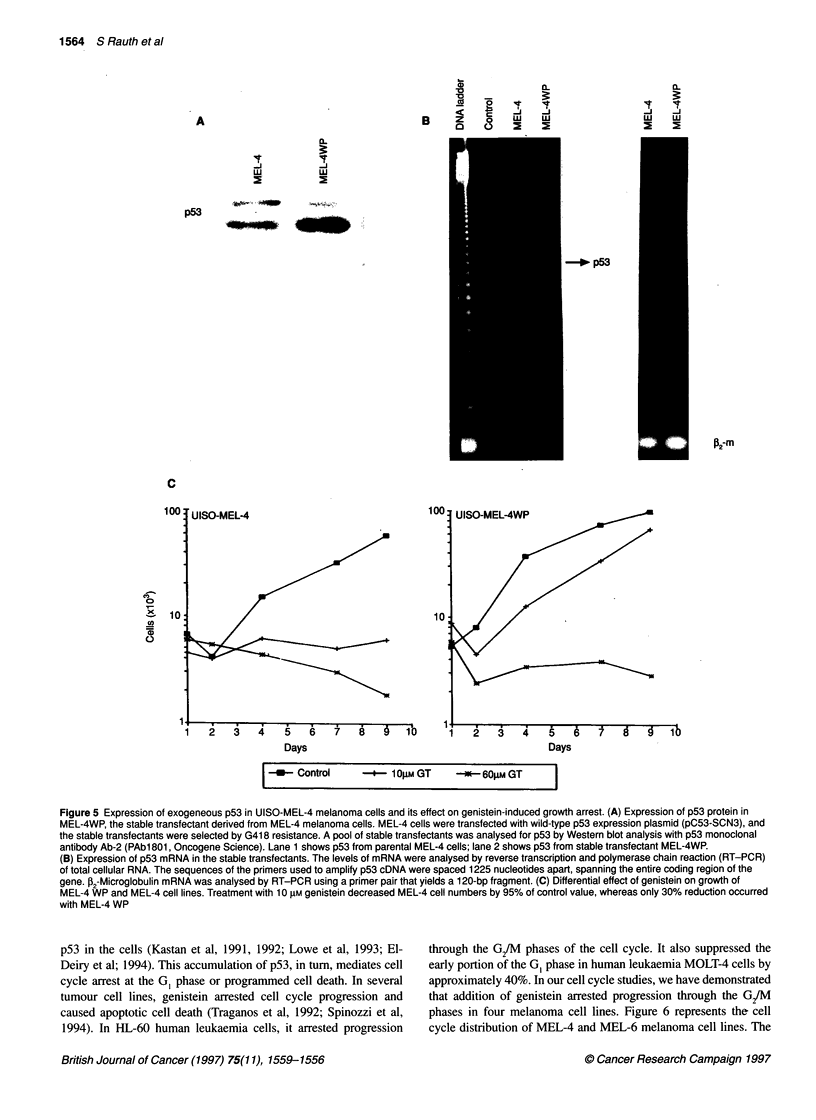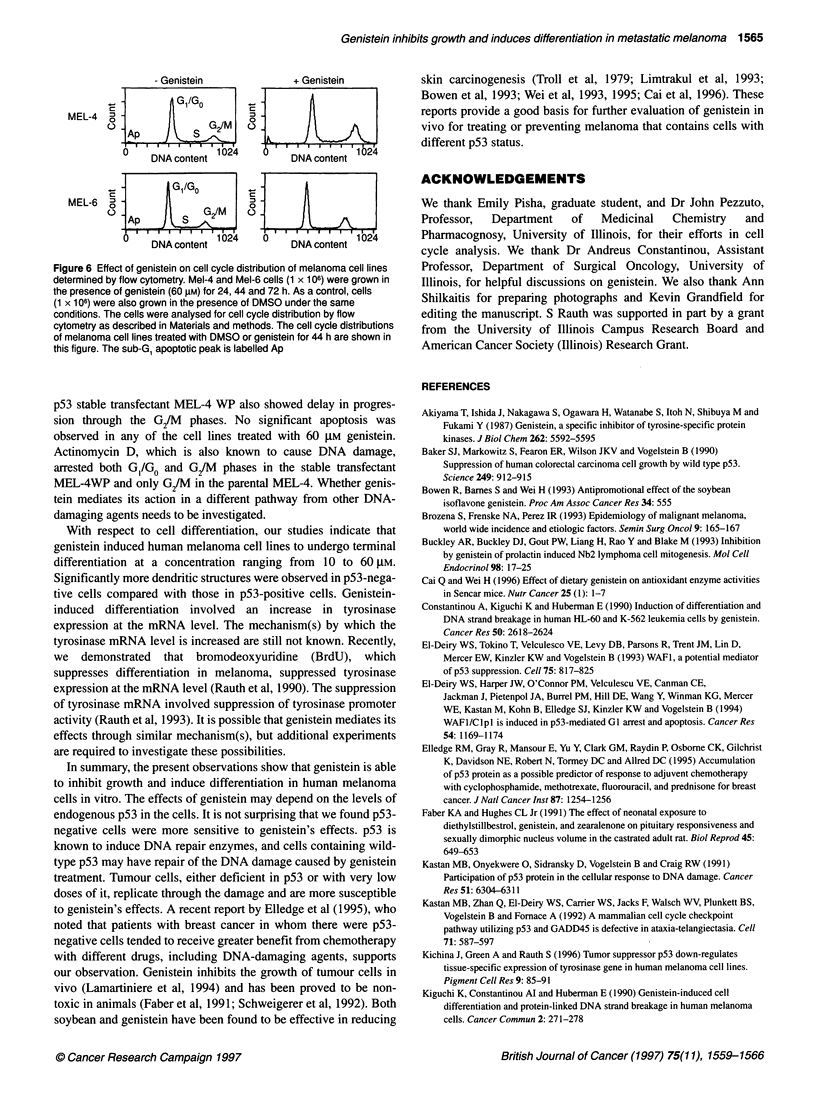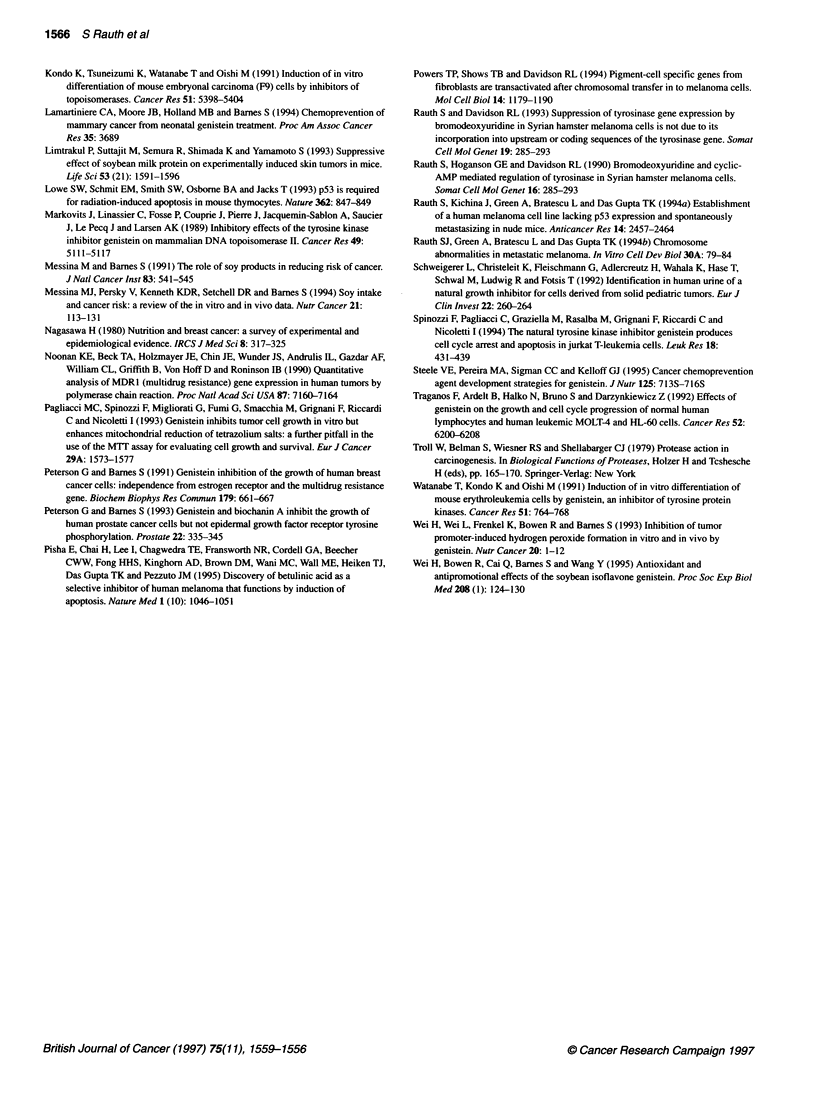# Inhibition of growth and induction of differentiation of metastatic melanoma cells in vitro by genistein: chemosensitivity is regulated by cellular p53.

**DOI:** 10.1038/bjc.1997.268

**Published:** 1997

**Authors:** S. Rauth, J. Kichina, A. Green

**Affiliations:** Department of Surgical Oncology, University of Illinois, Chicago 60612, USA.

## Abstract

**Images:**


British Journal of Cancer (1997) 75(11), 1559–1566
© 1997 Cancer Research Campaign

### Inhibition of growth and induction of differentiation of metastatic melanoma cells in vitro by genistein: chemosensitivity is regulated by cellular p53

**S Rauth[1,2], J Kichina[1,2] and A Green[1]**

[1]Departments of Surgical Oncology and [2]Genetics, University of Illinois, Chicago, IL, USA

**Summary** We have investigated the effect of the soybean isoflavone genistein on the growth and differentiation of human melanoma cells. Four human melanoma cell lines, either completely lacking or containing different levels of wild-type p53, were treated with genistein in vitro in culture. It has been found that genistein significantly inhibited cell growth and that the chemosensitivity might depend on cellular p53 content. Specifically, the data suggest that high levels of wild-type p53 expression make cells resistant to genistein's growth-inhibitory action. Further support for this observation came from the stable transfection studies in which p53 transfectants expressing high levels of wild-type p53 became resistant to genistein. With respect to cell differentiation, our study showed that genistein increased melanin content and tyrosinase activity and caused the cells to form dendrite-like structures. Cells lacking p53 responded more than cells with p53 to dendrite-like structure formation. We also observed that genistein-induced differentiation involved an increase in tyrosinase mRNA level; the mechanisms by which genistein increases tyrosinase transcripts remain to be elucidated. Genistein treatment of the melanoma cell lines resulted in cell cycle arrest at $G_2$/M check point and no significant apoptosis was observed.

**Keywords:** metastatic melanoma; growth; differentiation; p53; genistein

Currently, the incidence of malignant melanoma is on the rise. The increase in the number of melanomas diagnosed has been found to be greater than for any other cancer except lung cancer in women (Brozena et al, 1993). Melanomas are readily treatable during the early stages of development, but the prognosis is grave once the disease metastasizes. Currently available therapies are not effective in preventing or curing metastatic spread and morbidity in patients with this cancer. Better therapeutic and preventive approaches need to be developed against growth and metastasis of melanoma.

Epidemiological studies suggest that soy consumption may contribute to lower rates of cancers (Nagasawa et al, 1980; Messina and Barnes, 1991; Messina et al, 1994). One of the hypothesized candidates against malignancy in soybeans is genistein (4′,5,7-trihydroxyisoflavone), a major isoflavone of soybean. Several classes of compounds in soybeans, like protease inhibitors, saponins, phytosterols, and isoflavones, have demonstrated anti-cancer activity. The most recent experimental studies, however, suggest that genistein may play a major role in mediating the anti-carcinogenic effects of soybeans (Messina and Barnes, 1991). Purified genistein inhibited the growth of a wide range of cultured cancer cells like leukaemia, breast and prostate cancer, and lymphoma (Constantinou et al, 1990; Kondo et al, 1991; Watanabe et al, 1991; Peterson and Barnes, 1991, 1993; Pagliacci et al, 1993; Buckley et al, 1993). In vivo studies also demonstrated that genistein reduced both the incidence and the multiplicity of DMBA-induced mammary tumours and azoxymethane-induced colon tumours in rats (Lamartiniere et al, 1994; Steele et al, 1995). Among other enzymes, genistein inhibits the activity of protein tyrosine kinase (PTK) and topoisomerase II (Akiyama et al, 1987; Markovits et al, 1989). Mechanistically, genistein treatment caused DNA strand breakage, and DNA breaks were found to be protein linked (Constantinou et al, 1990; Kiguchi et al, 1990). Under certain treatment conditions, genistein caused cell cycle arrest and apoptotic cell death in several leukaemia cell lines (Traganos et al, 1992; Spinozzi et al, 1994). The precise molecular mechanism(s) by which genistein exerts its effects against tumour cells are still not clear.

Recent studies show that both soy and genistein are effective against skin cancer. Consumption of soybean diets delayed the onset of skin tumours induced by nitroquinoline-*N*-oxide and 12-*O*-tetradecanoyphorbol-13-acetate (TPA) (Troll et al, 1979). In a two-stage mouse skin carcinogenesis experiment, the soybean milk protein reduced both the incidence and the volume of skin tumours (Limtrakul et al, 1993). Topically applied genistein has been found to reduce the number of skin carcinomas induced by DMBA and TPA in CD1 mice (Bowen et al, 1993). The topical application of genistein also inhibited TPA-induced increases in hydrogen peroxide production in a mouse skin model (Wei et al, 1993, 1995). A more recent study showed that dietary genistein significantly enhanced antioxidant enzyme activities in skin (Cai et al, 1996).

In the present study, we examined the effect of genistein on the growth and differentiation of melanoma that arises from normal melanocytes commonly located in skin. We used four human melanoma cell lines that either completely lack or contain different levels of wild-type p53 as described earlier (Rauth et al, 1994*a*, *b*). The differentiated state of the cells was characterized morphologically and by tyrosinase gene expression and melanin content. The results showed that genistein significantly inhibited

*Received 18 June 1996*
*Revised 20 November 1996*
*Accepted 4 December 1996*

*Correspondence to:* S Rauth, Department of Surgical Oncology (M/C 820), 840 South Wood Street, Chicago, IL 60612, USA

growth and induced differentiation of human melanoma in culture. The results also suggested that the chemosensitivity of the cells might depend on cellular p53 content. Cells either lacking or containing low p53 protein levels were more sensitive to genistein-induced growth arrest than cells with high p53. Further support for these observations came from gene transfer studies, in which stable transfectants expressing high levels of wild-type p53 became resistant to genistein's growth-inhibitory action. With respect to differentiation, we found that melanoma cells not expressing p53 were more sensitive to genistein-induced dendrite-like structure formation than cells expressing high p53. Our study also showed that genistein-induced differentiation was associated with an increase in tyrosinase mRNA level. The mechanism(s) by which genistein increases tyrosinase transcripts remain(s) to be elucidated. Genistein treatment of the melanoma cell lines resulted in arrest of $G_2$/M check point of cell cycle distribution and no significant apoptosis was observed.

### MATERIALS AND METHODS

### Cells and media

The human melanoma cell lines, UISO-MEL-6, UISO-MEL-4, UISO MEL-7 and UISO-MEL-8, established and characterized in our laboratory (Rauth et al, 1994*a, b*), have been used in the present study. All cells were maintained in MEM-H [minimum essential medium with Hanks' balanced salt solution (Gibco, Grand Island, NY, USA)] containing fetal calf serum (2%), L-glutamine (1%), non-essential amino acids and penicillin–streptomycin (0.2%).

### Plasmids and reagents

The human wild-type p53 expression plasmid, pC53-SN3, and the expression vector without p53 cDNA, pCMVNeo, originally constructed by Bert Vogelstein (Baker et al, 1990), were obtained from Professor Kiranur Subramanian. Department of Genetics, University of Illinois, Chicago, USA. Genistein was obtained from Sigma Chemical Co.

### Transfection experiments

To isolate cells stably transfected with human wild-type p53 expression plasmid pC53-SN3, UISO-MEL-4 cells were co-transfected with plasmids pC53-SN3 and pIRVGalNeo as described earlier (Rauth et al, 1993). Three days after transfection, the cells were subcultured in the presence of 1 mg $ml^{-1}$ G418 (Sigma Chemical Co.). The selective media with G418 was replaced every 3 days, and the colonies that appeared after 10–14 days were pooled and subcultured for further analysis. The stable transfectant was designated as UISO-MEL-4WP. As a control, cells stably transfected with the expression vector were also isolated similarly.

### Western blot analysis

The melanoma cell lines were analysed for p53 protein level by Western blot analysis. These cells were then lysed in lysis buffer containing 50 mM Tris (pH 8.0), 250 mM sodium chloride and 0.1% Nonidet p-40 as described (Kichina et al, 1996). The cell extracts containing equal amounts of protein (150 μg) were separated on a sodium dodecyl sulphate (SDS)–12.5% polyacrylamide gel. The proteins were transferred electrophoretically to a nitrocellular membrane, and the p53 proteins in the blot were detected with a 1:200 dilution of p53 monoclonal antibody Ab-2 (pAb1801, Oncogene Science) and the enhanced chemiluminescence system (Amersham).

### Tyrosinase expression by reverse transcription and polymerase chain reaction (RT–PCR)

The human melanoma cell lines were analysed for tyrosinase expression at the mRNA level by reverse transcription of the total cellular RNA and PCR amplification of the reverse-transcribed tyrosinase transcript using primers specific for human tyrosinase sequences. The PCR amplification of the reverse-transcribed RNA was performed in a 100-μl volume with 2.5 U of *Taq* DNA polymerase (Promega), using an Eri Comp DNA thermocycler following the procedure described previously (Kichina et al, 1996). The sequences of the primers used to amplify tyrosinase cDNA sequences were: primer 1: 5′-TAGGACCTGCCAGTTGCCTTTCT-3′ (sense); primer 2: 5′-AAGGCATTGTGCATGC-3′ (antisense). The sequences of the primers were obtained from the published report (Powers et al, 1994) and were synthesized on an ABI model 394 DNA synthesizer. The primer 1 is located on exon 1, whereas primer 2 is located on exon 2 of the tyrosinase gene, and these primers amplify a 840-bp cDNA fragment. The control PCR amplification reaction mixture contained the reagent mixture for PCR amplification without the added cDNA.

### p53 expression by RT–PCR

p53 mRNA was analysed by RT–PCR of total cellular RNA from the stable transfectants as described earlier (Kichina et al, 1996). The sequences of the primers used to amplify p53 cDNA are spaced 1225 nucleotides apart and yield a full-length cDNA fragment. Primer 1: 5′-AGACTGCCTTCCGGGTCACT-3′; primer 2: 5′-GGGAACAAAGAAGTGGAGAAT-3′. $\beta_2$-Microglobulin ($\beta_2$-m) mRNA, used as an internal control, was also analysed by RT–PCR. The sequences of the primers used to amplify $\beta_2$-microglobulin were obtained from published sequences (Noonan et al, 1990) and yielded a 120-bp cDNA fragment.

### Flow cytometry

Melanoma cells ($1 \times 10^7$) were cultured in the presence of dimethyl sulphoxide (DMSO) or genistein for 0, 24, 44 and 72 h. Cells were harvested, fixed with ethanol and flow cytometry was performed after treatment with trypsin and RNAase A as described previously (Pisha et al, 1995).

### RESULTS

### Differential effect of genistein on melanoma growth in vitro

To investigate genistein's effects on melanoma cells, we have used four cell lines (UISO-MEL-6, UISO-MEL-4, UISO-MEL-7 and UISO-MEL-8) that either lack or contain different levels of wild-type p53 (Rauth et al, 1994*a, b*). In our previous study, we have analysed ten melanoma cell lines for p53 alterations at the DNA, RNA and protein levels (Rauth et al, 1994*a*). Nucleotide sequencing demonstrated no mutations in exons 5 to 8 of the p53 gene in any of the cell lines tested. Northern blot analysis showed 60% of the cell lines expressed high levels of p53 mRNA.

 © Cancer Research Campaign 1997

**Figure 1** Western blot analysis for p53 protein levels in melanoma cell lines. Cell extracts containing equal amounts of protein (150 μg) were separated on a SDS–12.5% polyacrylamide gel. Proteins were transferred electrophoretically to a nitrocellulose membrane, and the p53 proteins in the blot were detected with a 1:200 dilution of p53 monoclonal antibody Ab-2 (pAb1801, Oncogene Science) and the enhanced chemiluminescence system (Amersham)

Immunocytochemical analysis using antibody specific for wild-type and mutant p53 protein agreed with the DNA and RNA analysis data. The cytogenetic analysis of these cell lines showed that 17p, where p53 is located, is missing in UISO-MEL-6 cell line owing to isochromosome formation (Rauth et al, 1994*b*). Taken together, we found that UISO-MEL-6 completely lacks and UISO-MEL-4 contains a very low level of wild-type p53 protein. On the other hand, UISO-MEL-7 and UISO-MEL-8 express moderate and high levels of wild-type p53 protein respectively. In the present study, the levels of p53 protein in the melanoma cell lines were quantified by Western blot analysis. As shown in Figure 1, different levels of p53 protein are present in four cell lines that agree with our previous data. To test the functionality of endogenous wild-type p53 protein in these cell lines, a number of experiments using transient transfection approaches and a construct containing CAT reporter gene ligated to p53-responsive p21 promoter sequences have been done. Our preliminary experiments showed that endogenous p53 in these cell lines might be functional and could induce CAT gene expression from a p53-responsive element (data not shown).

The four cell lines, either lacking or containing various p53 levels, were treated with different concentrations of genistein, and the cell numbers were counted for 9 days (renewing the culture with fresh media every 3 days). As shown in Figure 2, genistein significantly inhibited the growth of melanoma cells. The growth rates of UISO-MEL-6 and UISO-MEL-4, which lack and contain very low wild-type p53 respectively, were very sensitive to genistein. A concentration of genistein as low as 10 μM significantly inhibited the growth of those cells in culture. In contrast, UISO-MEL-7 (moderate p53) and UISO-MEL-8 (high p53) were less sensitive to genistein. UISO-MEL-8 cells, which contain a high level of wild-type p53, were considerably resistant to genistein's growth-inhibitory effects. With 10–60 μM genistein, those cells

**Figure 2** Effect of genistein (GT) on growth of four human melanoma cell lines containing different levels of p53. Melanoma cells growing exponentially were plated in triplicate at a density of 1 × $10^4$ cells per well in multiwell dishes with MEM-E and 10% fetal bovine serum (FBS), and cultured for 9 days in the absence and presence of genistein. Cultured media were renewed every 3 days

© Cancer Research Campaign 1997

**Figure 3** Increase in tyrosinase mRNA expression in UISO-MEL-7 cells treated for 6 days with 10 μM and 30 μM genistein. Tyrosinase mRNA was analysed by reverse transcription and polymerase chain reaction (RT–PCR). Different volumes of RT–PCR mix (2, 4 and 8 μl) of each sample was loaded on 1% agarose gel. Lanes 1, 2 and 3, untreated cells; lanes 4, 5 and 6, 10 μM genistein-treated cells; lanes 7, 8 and 9, 30 μM genistein-treated cells grew almost at the same rate as the untreated cells. These data suggest that genistein inhibits melanoma growth in vitro and that growth inhibition is more pronounced in cell lines either lacking or with low p53.

### Genistein induces differentiation and activates tyrosinase expression at the mRNA level

Genistein's effects on the differentiation of melanoma cell lines containing different levels of p53 were examined. The induction of differentiation and acquiring of mature phenotype were characterized by the production of melanin, increase in tyrosinase expression and formation of dendrite-like cellular protrusions. In culture, cells were treated with 10 μM and 60 μM genistein for 9 days, and cells were analysed for changes in tyrosinase expression and morphology. This study demonstrated a dose- and and time-dependent increase in pigmentation (visually detectable) and tyrosinase activity (analysed by the DOPA method, data not shown). The increase in tyrosinase activity was associated with an increase in the tyrosinase mRNA level. The tyrosinase mRNA was analysed by reverse transcription and polymerase chain reaction (RT–PCR) using tyrosinase gene-specific primers, which yielded an 840-bp tyrosinase cDNA fragment. As shown in Figure 3, increased levels of tyrosinase transcripts were detected in UISO-MEL-7 melanoma cells following treatment with 10 μM and 30 μM genistein for 6 days. $\beta_2$-Microglobulin mRNA, used as an internal control, remained unchanged in the genistein-treated cells (data not shown). These results indicate that genistein induces differentiation and enhances tyrosinase expression at the mRNA level.

The differentiated state of melanoma cells was then monitored by the formation of dendrite-like cellular protrusions. The melanoma cell lines containing different levels of p53 were treated with genistein of different concentrations, and the cells were examined for changes in morphology. As shown in Figure 4, the treatment of melanoma cell lines, MEL-6 (p53 negative) and MEL-7 (p53 positive), with 10 μM and 60 μM genistein caused the cells to form dendrite-like structures. These dendritic structures became progressively longer, and by 6 days after treatment, more than 90% of the cells showed an extensive network of such structures. The cell lines either lacking p53 or with low p53 became more dendritic than those containing a high level of p53.

### Effect of exogenous p53 on chemosensitivity

To analyse the relationship between genistein's growth-suppression function and p53 levels of the cells, we made a stable melanoma cell line overexpressing wild-type p53. UISO MEL-4, containing a low level of wild-type p53, was stably transfected with wild-type p53 expression plasmid (pC53-SCN3), and the stable transfectants were selected by G418 resistance. As a control, the expression vector without p53 cDNA insert (pCMVNeo) was also stably transfected into the same line under the same conditions. The number of G418-resistant colonies obtained with the wild-type p53 was found to be slightly lower than that obtained with the expression vector. UISO-MEL-6, which completely lacks p53, was also transfected with the wild-type p53 expression plasmid under the same conditions, but no G418-resistant colonies with wild-type p53 survived.

A pool of UISO-MEL-4 stable transfectants was analysed for p53 protein level by Western blot analysis with p53 monoclonal antibody Ab-2 (PAb1801, Oncogene Science). As shown in Figure 5A, the stable transfectants, MEL-4 WP, expressed a high level of p53 protein compared with the parental MEL-4 cells.

To determine whether p53 mRNA is transcribed from the transfected plasmid, transfectants were analysed for p53 mRNA levels by reverse transcription and polymerase chain reaction. As shown in Figure 5B, increased levels of p53 mRNA were detected in the stable transfectants compared with untransfected cells. $\beta_2$-Microglobulin ($\beta_2$-m) mRNA, used as an internal control, remained unchanged in the transfectants.

To test the functionality of wild-type p53 in the stable transfectant, we have examined the levels of p21 mRNA. Expression of p21 gene is known to be induced directly by wild-type p53 (El-Diery et al, 1993). Our study showed that the p53 stable transfectants expressed a high level of p21 mRNA or protein (data not shown). We have also tested whether wild-type p53 in the stable transfectant could induce CAT gene expression from p53-responsive p21 promoter sequences in transient transfection assays. Our preliminary data showed that the stable transfectants demonstrated a higher level of CAT activity compared with that in the parental MEL-4 cells (data not shown). These results suggested that p53 in the stable transfectant was wild-type and functional.

To examine genistein's effects on the growth of the stable transfectants, cells were treated with 10 μM and 60 μM genistein in culture. As shown in Figure 5C, the stable transfectants overexpressing p53 became resistant to genistein at the 10 μM concentration. Only a small decrease in the growth rate of stable transfectant, MEL-4 Wp, was detected, whereas parental MEL-4 cells demonstrated significant growth inhibition following genistein treatment. These results suggest that expression of high levels of wild-type p53 make melanoma cells resistant to genistein-induced growth arrest.

To determine whether genistein treatment of melanoma cells resulted in alteration of cell cycle progression, the cell cycle patterns of the melanoma cell lines were examined by flow cytometric analysis. At a 60 μM concentration, genistein arrested $G_2/M$ phase in all four cell lines. Figure 6 shows cell cycle dynamics of MEL-6 and MEL-4 cells untreated or treated with genistein. At a lower concentration of genistein (30 μM), small changes in the cell cycle distribution at $G_2/M$ phase were observed. The stable transfectant, MEL-4 WP, also showed delay in $G_2/M$ phase. Actinomycin D (1 ng $ml^{-1}$) treatment, on the other hand, showed delay in both $G_1/G_0$ and $G_2/M$ phases in MEL-4 WP and $G_2/M$ in parental MEL-4 cells (data not shown). Only a very small

© Cancer Research Campaign 1997

**Figure 4** Dendrite-like structure formation in UISO-MEL-6 (p53 negative) and UISO-MEL-7 (p53 positive) cells treated for 6 days with 10 μM and 60 μM genistein. (**A–C**) UISO-MEL-6 cells; (**D–F**) UISO-MEL-7 cells. Cell morphology was visualized by light microscopy. (**A** and **D**) untreated cells; (**B** and **E**) 10 μM genistein-treated cells; (**C** and **F**) 60μM genistein-treated cells apoptotic peak was detected in genistein (60 μM)-treated melanoma cells analysed flow cytometrically.

### DISCUSSION

The data presented in this report demonstrate that genistein can arrest growth and enhance differentiation of metastatic melanoma cells; more importantly, the chemosensitivity of these cells may be regulated by cellular p53. We used four cell lines that have been well characterized for p53 at DNA, RNA and protein levels (Rauth et al, 1994*a*, *b*). The results presented here showed that the growth of cells either negative (UISO-MEL-6) or low (UISO-MEL-4) in wild-type p53 was almost completely inhibited when treated with genistein. In contrast, only a small decrease in growth rate was observed in melanoma cells containing a high level of wild-type p53.

The melanoma cell lines were originally derived from the biopsies of different patients (Rauth et al, 1994*b*) and may differ in factors besides the endogenous p53 content. To investigate whether the differential effect of genistein on cell growth is related to the levels of endogenous p53, we stably transfected the melanoma cell line MEL-4 with wild-type p53 expression plasmid. The functionality of wild-type p53 expressed in the stable transfectant was tested by an increase in endogenous p21 expression. To determine the effects of genistein, parental cells containing very low levels of endogenous wild-type p53, and the transfectants expressing high levels of p53, were treated with different doses of genistein. The data presented in Figure 5 clearly show that p53 transfectants became significantly less sensitive than the parental cells to genistein's growth-inhibitory effects.

As a corollary to our observations, a recent study reported that genistein induced endogenous p53 levels in several tumour cell lines (El-Deiry et al, 1994). Previous studies have demonstrated that genistein caused single- and double-strand DNA breaks in tumour cells (Constantinou et al, 1990; Kiguchi et al, 1990). Many cancer chemotherapeutic drugs and ultraviolet light or gamma-irradiation, which damage DNA, are known to induce accumulation of normal

© Cancer Research Campaign 1997

**Figure 5** Expression of exogeneous p53 in UISO-MEL-4 melanoma cells and its effect on genistein-induced growth arrest. (**A**) Expression of p53 protein in MEL-4WP, the stable transfectant derived from MEL-4 melanoma cells. MEL-4 cells were transfected with wild-type p53 expression plasmid (pC53-SCN3), and the stable transfectants were selected by G418 resistance. A pool of stable transfectants was analysed for p53 by Western blot analysis with p53 monoclonal antibody Ab-2 (PAb1801, Oncogene Science). Lane 1 shows p53 from parental MEL-4 cells; lane 2 shows p53 from stable transfectant MEL-4WP.
(**B**) Expression of p53 mRNA in the stable transfectants. The levels of mRNA were analysed by reverse transcription and polymerase chain reaction (RT–PCR) of total cellular RNA. The sequences of the primers used to amplify p53 cDNA were spaced 1225 nucleotides apart, spanning the entire coding region of the gene. $\beta_2$-Microglobulin mRNA was analysed by RT–PCR using a primer pair that yields a 120-bp fragment. (**C**) Differential effect of genistein on growth of MEL-4 WP and MEL-4 cell lines. Treatment with 10 μM genistein decreased MEL-4 cell numbers by 95% of control value, whereas only 30% reduction occurred with MEL-4 WP

p53 in the cells (Kastan et al, 1991, 1992; Lowe et al, 1993; El-Deiry et al; 1994). This accumulation of p53, in turn, mediates cell cycle arrest at the $G_1$ phase or programmed cell death. In several tumour cell lines, genistein arrested cell cycle progression and caused apoptotic cell death (Traganos et al, 1992; Spinozzi et al, 1994). In HL-60 human leukaemia cells, it arrested progression through the $G_2$/M phases of the cell cycle. It also suppressed the early portion of the $G_1$ phase in human leukaemia MOLT-4 cells by approximately 40%. In our cell cycle studies, we have demonstrated that addition of genistein arrested progression through the $G_2$/M phases in four melanoma cell lines. Figure 6 represents the cell cycle distribution of MEL-4 and MEL-6 melanoma cell lines. The

 © Cancer Research Campaign 1997

**Figure 6** Effect of genistein on cell cycle distribution of melanoma cell lines determined by flow cytometry. Mel-4 and Mel-6 cells ($1 \times 10^6$) were grown in the presence of genistein (60 μM) for 24, 44 and 72 h. As a control, cells ($1 \times 10^6$) were also grown in the presence of DMSO under the same conditions. The cells were analysed for cell cycle distribution by flow cytometry as described in Materials and methods. The cell cycle distributions of melanoma cell lines treated with DMSO or genistein for 44 h are shown in this figure. The sub-$G_1$ apoptotic peak is labelled Ap p53 stable transfectant MEL-4 WP also showed delay in progression through the $G_2/M$ phases. No significant apoptosis was observed in any of the cell lines treated with 60 μM genistein. Actinomycin D, which is also known to cause DNA damage, arrested both $G_1/G_0$ and $G_2/M$ phases in the stable transfectant MEL-4WP and only $G_2/M$ in the parental MEL-4. Whether genistein mediates its action in a different pathway from other DNA-damaging agents needs to be investigated.

With respect to cell differentiation, our studies indicate that genistein induced human melanoma cell lines to undergo terminal differentiation at a concentration ranging from 10 to 60 μM. Significantly more dendritic structures were observed in p53-negative cells compared with those in p53-positive cells. Genistein-induced differentiation involved an increase in tyrosinase expression at the mRNA level. The mechanism(s) by which the tyrosinase mRNA level is increased are still not known. Recently, we demonstrated that bromodeoxyuridine (BrdU), which suppresses differentiation in melanoma, suppressed tyrosinase expression at the mRNA level (Rauth et al, 1990). The suppression of tyrosinase mRNA involved suppression of tyrosinase promoter activity (Rauth et al, 1993). It is possible that genistein mediates its effects through similar mechanism(s), but additional experiments are required to investigate these possibilities.

In summary, the present observations show that genistein is able to inhibit growth and induce differentiation in human melanoma cells in vitro. The effects of genistein may depend on the levels of endogenous p53 in the cells. It is not surprising that we found p53-negative cells were more sensitive to genistein's effects. p53 is known to induce DNA repair enzymes, and cells containing wild-type p53 may have repair of the DNA damage caused by genistein treatment. Tumour cells, either deficient in p53 or with very low doses of it, replicate through the damage and are more susceptible to genistein's effects. A recent report by Elledge et al (1995), who noted that patients with breast cancer in whom there were p53-negative cells tended to receive greater benefit from chemotherapy with different drugs, including DNA-damaging agents, supports our observation. Genistein inhibits the growth of tumour cells in vivo (Lamartiniere et al, 1994) and has been proved to be non-toxic in animals (Faber et al, 1991; Schweigerer et al, 1992). Both soybean and genistein have been found to be effective in reducing skin carcinogenesis (Troll et al, 1979; Limtrakul et al, 1993; Bowen et al, 1993; Wei et al, 1993, 1995; Cai et al, 1996). These reports provide a good basis for further evaluation of genistein in vivo for treating or preventing melanoma that contains cells with different p53 status.

### ACKNOWLEDGEMENTS

We thank Emily Pisha, graduate student, and Dr John Pezzuto, Professor, Department of Medicinal Chemistry and Pharmacognosy, University of Illinois, for their efforts in cell cycle analysis. We thank Dr Andreus Constantinou, Assistant Professor, Department of Surgical Oncology, University of Illinois, for helpful discussions on genistein. We also thank Ann Shilkaitis for preparing photographs and Kevin Grandfield for editing the manuscript. S Rauth was supported in part by a grant from the University of Illinois Campus Research Board and American Cancer Society (Illinois) Research Grant.

### REFERENCES

Akiyama T, Ishida J, Nakagawa S, Ogawara H, Watanabe S, Itoh N, Shibuya M and Fukami Y (1987) Genistein, a specific inhibitor of tyrosine-specific protein kinases. *J Biol Chem* **262**: 5592–5595

Baker SJ, Markowitz S, Fearon ER, Wilson JKV and Vogelstein B (1990) Suppression of human colorectal carcinoma cell growth by wild type p53. *Science* **249**: 912–915

Bowen R, Barnes S and Wei H (1993) Antipromotional effect of the soybean isoflavone genistein. *Proc Am Assoc Cancer Res* **34**: 555

Brozena S, Frenske NA, Perez IR (1993) Epidemiology of malignant melanoma, world wide incidence and etiologic factors. *Semin Surg Oncol* **9**: 165–167

Buckley AR, Buckley DJ, Gout PW, Liang H, Rao Y and Blake M (1993) Inhibition by genistein of prolactin induced Nb2 lymphoma cell mitogenesis. *Mol Cell Endocrinol* **98**: 17–25

Cai Q and Wei H (1996) Effect of dietary genistein on antioxidant enzyme activities in Sencar mice. *Nutr Cancer* **25** (1): 1–7

Constantinou A, Kiguchi K and Huberman E (1990) Induction of differentiation and DNA strand breakage in human HL-60 and K-562 leukemia cells by genistein. *Cancer Res* **50**: 2618–2624

El-Deiry WS, Tokino T, Velculesco VE, Levy DB, Parsons R, Trent JM, Lin D, Mercer EW, Kinzler KW and Vogelstein B (1993) WAF1, a potential mediator of p53 suppression. *Cell* **75**: 817–825

El-Deiry WS, Harper JW, O'Connor PM, Velculescu VE, Canman CE, Jackman J, Pietenpol JA, Burrel PM, Hill DE, Wang Y, Winman KG, Mercer WE, Kastan M, Kohn B, Elledge SJ, Kinzler KW and Vogelstein B (1994) WAF1/C1p1 is induced in p53-mediated G1 arrest and apoptosis. *Cancer Res* **54**: 1169–1174

Elledge RM, Gray R, Mansour E, Yu Y, Clark GM, Raydin P, Osborne CK, Gilchrist K, Davidson NE, Robert N, Tormey DC and Allred DC (1995) Accumulation of p53 protein as a possible predictor of response to adjuvent chemotherapy with cyclophosphamide, methotrexate, fluorouracil, and prednisone for breast cancer. *J Natl Cancer Inst* **87**: 1254–1256

Faber KA and Hughes CL Jr (1991) The effect of neonatal exposure to diethylstillbestrol, genistein, and zearalenone on pituitary responsiveness and sexually dimorphic nucleus volume in the castrated adult rat. *Biol Reprod* **45**: 649–653

Kastan MB, Onyekwere O, Sidransky D, Vogelstein B and Craig RW (1991) Participation of p53 protein in the cellular response to DNA damage. *Cancer Res* **51**: 6304–6311

Kastan MB, Zhan Q, El-Deiry WS, Carrier WS, Jacks F, Walsch WV, Plunkett BS, Vogelstein B and Fornace A (1992) A mammalian cell cycle checkpoint pathway utilizing p53 and GADD45 is defective in ataxia-telangiectasia. *Cell* **71**: 587–597

Kichina J, Green A and Rauth S (1996) Tumor suppressor p53 down-regulates tissue-specific expression of tyrosinase gene in human melanoma cell lines. *Pigment Cell Res* **9**: 85–91

Kiguchi K, Constantinou AI and Huberman E (1990) Genistein-induced cell differentiation and protein-linked DNA strand breakage in human melanoma cells. *Cancer Commun* **2**: 271–278

© Cancer Research Campaign 1997

Kondo K, Tsuneizumi K, Watanabe T and Oishi M (1991) Induction of in vitro differentiation of mouse embryonal carcinoma (F9) cells by inhibitors of topoisomerases. *Cancer Res* **51**: 5398–5404

Lamartiniere CA, Moore JB, Holland MB and Barnes S (1994) Chemoprevention of mammary cancer from neonatal genistein treatment. *Proc Am Assoc Cancer Res* **35**: 3689

Limtrakul P, Suttajit M, Semura R, Shimada K and Yamamoto S (1993) Suppressive effect of soybean milk protein on experimentally induced skin tumors in mice. *Life Sci* **53** (21): 1591–1596

Lowe SW, Schmit EM, Smith SW, Osborne BA and Jacks T (1993) p53 is required for radiation-induced apoptosis in mouse thymocytes. *Nature* **362**: 847–849

Markovits J, Linassier C, Fosse P, Couprie J, Pierre J, Jacquemin-Sablon A, Saucier J, Le Pecq J and Larsen AK (1989) Inhibitory effects of the tyrosine kinase inhibitor genistein on mammalian DNA topoisomerase II. *Cancer Res* **49**: 5111–5117

Messina M and Barnes S (1991) The role of soy products in reducing risk of cancer. *J Natl Cancer Inst* **83**: 541–545

Messina MJ, Persky V, Kenneth KDR, Setchell DR and Barnes S (1994) Soy intake and cancer risk: a review of the in vitro and in vivo data. *Nutr Cancer* **21**: 113–131

Nagasawa H (1980) Nutrition and breast cancer: a survey of experimental and epidemiological evidence. *IRCS J Med Sci* **8**: 317–325

Noonan KE, Beck TA, Holzmayer JE, Chin JE, Wunder JS, Andrulis IL, Gazdar AF, William CL, Griffith B, Von Hoff D and Roninson IB (1990) Quantitative analysis of MDR1 (multidrug resistance) gene expression in human tumors by polymerase chain reaction. *Proc Natl Acad Sci USA* **87**: 7160–7164

Pagliacci MC, Spinozzi F, Migliorati G, Fumi G, Smacchia M, Grignani F, Riccardi C and Nicoletti I (1993) Genistein inhibits tumor cell growth in vitro but enhances mitochondrial reduction of tetrazolium salts: a further pitfall in the use of the MTT assay for evaluating cell growth and survival. *Eur J Cancer* **29A**: 1573–1577

Peterson G and Barnes S (1991) Genistein inhibition of the growth of human breast cancer cells: independence from estrogen receptor and the multidrug resistance gene. *Biochem Biophys Res Commun* **179**: 661–667

Peterson G and Barnes S (1993) Genistein and biochanin A inhibit the growth of human prostate cancer cells but not epidermal growth factor receptor tyrosine phosphorylation. *Prostate* **22**: 335–345

Pisha E, Chai H, Lee I, Chagwedra TE, Fransworth NR, Cordell GA, Beecher CWW, Fong HHS, Kinghorn AD, Brown DM, Wani MC, Wall ME, Heiken TJ, Das Gupta TK and Pezzuto JM (1995) Discovery of betulinic acid as a selective inhibitor of human melanoma that functions by induction of apoptosis. *Nature Med* **1** (10): 1046–1051

Powers TP, Shows TB and Davidson RL (1994) Pigment-cell specific genes from fibroblasts are transactivated after chromosomal transfer in to melanoma cells. *Mol Cell Biol* **14**: 1179–1190

Rauth S and Davidson RL (1993) Suppression of tyrosinase gene expression by bromodeoxyuridine in Syrian hamster melanoma cells is not due to its incorporation into upstream or coding sequences of the tyrosinase gene. *Somat Cell Mol Genet* **19**: 285–293

Rauth S, Hoganson GE and Davidson RL (1990) Bromodeoxyuridine and cyclic-AMP mediated regulation of tyrosinase in Syrian hamster melanoma cells. *Somat Cell Mol Genet* **16**: 285–293

Rauth S, Kichina J, Green A, Bratescu L and Das Gupta TK (1994*a*) Establishment of a human melanoma cell line lacking p53 expression and spontaneously metastasizing in nude mice. *Anticancer Res* **14**: 2457–2464

Rauth SJ, Green A, Bratescu L and Das Gupta TK (1994*b*) Chromosome abnormalities in metastatic melanoma. *In Vitro Cell Dev Biol* **30A**: 79–84

Schweigerer L, Christeleit K, Fleischmann G, Adlercreutz H, Wahala K, Hase T, Schwal M, Ludwig R and Fotsis T (1992) Identification in human urine of a natural growth inhibitor for cells derived from solid pediatric tumors. *Eur J Clin Invest* **22**: 260–264

Spinozzi F, Pagliacci C, Graziella M, Rasalba M, Grignani F, Riccardi C and Nicoletti I (1994) The natural tyrosine kinase inhibitor genistein produces cell cycle arrest and apoptosis in jurkat T-leukemia cells. *Leuk Res* **18**: 431–439

Steele VE, Pereira MA, Sigman CC and Kelloff GJ (1995) Cancer chemoprevention agent development strategies for genistein. *J Nutr* **125**: 713S–716S

Traganos F, Ardelt B, Halko N, Bruno S and Darzynkiewicz Z (1992) Effects of genistein on the growth and cell cycle progression of normal human lymphocytes and human leukemic MOLT-4 and HL-60 cells. *Cancer Res* **52**: 6200–6208

Troll W, Belman S, Wiesner RS and Shellabarger CJ (1979) Protease action in carcinogenesis. In *Biological Functions of Proteases*, Holzer H and Tcshesche H (eds), pp. 165–170. Springer-Verlag: New York

Watanabe T, Kondo K and Oishi M (1991) Induction of in vitro differentiation of mouse erythroleukemia cells by genistein, an inhibitor of tyrosine protein kinases. *Cancer Res* **51**: 764–768

Wei H, Wei L, Frenkel K, Bowen R and Barnes S (1993) Inhibition of tumor promoter-induced hydrogen peroxide formation in vitro and in vivo by genistein. *Nutr Cancer* **20**: 1–12

Wei H, Bowen R, Cai Q, Barnes S and Wang Y (1995) Antioxidant and antipromotional effects of the soybean isoflavone genistein. *Proc Soc Exp Biol Med* **208** (1): 124–130

*© Cancer Research Campaign 1997*